# Application of 3D Hepatic Plate-Like Liver Model for Statin-Induced Hepatotoxicity Evaluation

**DOI:** 10.3389/fbioe.2022.826093

**Published:** 2022-03-17

**Authors:** Jiecheng Xu, Daogang Pan, Wei Liao, Zhidong Jia, Mingxin Pan, Jun Weng, Xu Han, Shao Li, Yang Li, Kangyan Liang, Shuqin Zhou, Qing Peng, Yi Gao

**Affiliations:** ^1^ Department of Hepatobiliary Surgery II, Guangdong Provincial Research Center for Artificial Organ and Tissue Engineering, Guangzhou Clinical Research and Transformation Center for Artificial Liver, Institute of Regenerative Medicine, Zhujiang Hospital of Southern Medical University, Guangzhou, China; ^2^ Guangzhou Overseas Chinese Hospital, The First Affiliated Hospital of Jinan University, Guangzhou, China; ^3^ Department of Anesthesiology, Zhujiang Hospital, Southern Medical University, Guangzhou, China; ^4^ State Key Laboratory of Organ Failure Research, Southern Medical University, Guangzhou, China

**Keywords:** 3D culture, hydrogel, hepatic plate, polarity, drug-induced liver injury (DILI), statins

## Abstract

**Background:** Drug-induced liver injury is one of the main reasons of withdrawals of drugs in postmarketing stages. However, an experimental model(s) which can accurately recapitulates liver functions and reflects the level of drug hepatotoxicity is lack. In this study, we assessed drug hepatotoxicity using a novel three-dimensional hepatic plate-like hydrogel fiber (3D-P) co-culture system.

**Methods:** During the 28-days culture period, the liver-specific functions, hepatocyte polarity, sensitivity of drug-induced toxicity of 3D-P co-culture system were evaluated with 2D co-culture, collagen sandwich co-culture, 3D hybrid hydrogel fiber co-culture and human primary hepatocytes as controls. High-content imaging and analysis (HCA) methods were used to explore the hepatotoxicity mechanism of five statins.

**Results:** The 3D-P co-culture system showed enhancing liver-specific functions, cytochrome P450 enzymes (CYPs) metabolic activity and bile excretion, which were considered to result from improved hepatocyte polarity. Three of the statins may cause acute or chronic hepatotoxicity by *via* different mechanisms, such as cholestatic liver injury.

**Conclusion:** Our 3D-P co-culture system is characterized by its biomimetic hepatic plate-like structure, long-term stable liver specificity, and prominent bile secretion function, making it applicable for acute/chronic drug hepatotoxicity assessments.

## Introduction

**FIGURE 1:**
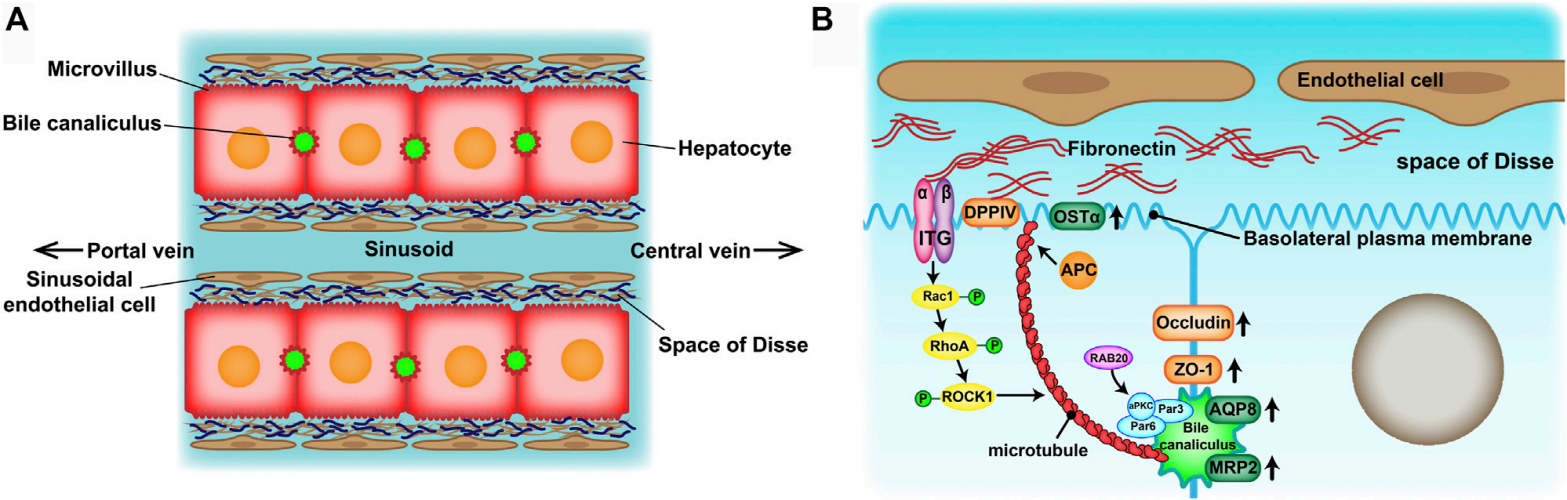
**(A)** Structure of the hepatic plates in the liver lobule. Hepatocytes are arranged in cord-like structures and separated from the sinusoid with a thickness of one hepatocyte layer. The hepatic plates are surrounded by sinusoidal endothelial cell layers through the space of Disse. **(B)** Mechanisms of apico-basal polarity formation. Signals from the extracellular matrix (ECM)–cell interaction may induce cytoskeletal reorganization and cell polarization to initiate basolateral plasma membrane and bile canaliculus formation.

Drug-induced liver injury (DILI) is the most frequent reason for the withdrawal of drugs from the market (Lasser et al., 2002) and has become one of the most challenging diseases worldwide (Shen et al., 2019; Bunchorntavakul and Reddy, 2017). Therefore, an accurate prediction of DILI in the early stages of drug development is crucial for reducing its incidence and drug development costs. At present, experimental animals and *in vitro* human hepatocyte cultures have been widely used in drug hepatotoxicity evaluation tests. However, there are many inconveniences of experimental animals such as species specificity of metabolic enzymatic activities, animal welfare and constraints of cost. Because of the convenience of application, two-dimensional (2D)-cultured human hepatocytes are still dominant in drug hepatotoxicity evaluation. However, their drug response may not reflect the actual response of hepatocytes *in vivo* (Fang and Eglen, 2017). Culturing hepatocytes in a conventional 2D system causes morphological alterations that are provoked by an epithelial-mesenchymal transition (EMT) (Liu et al., 2018). Eventually, the hepatocytes lose their specific liver functions and cell polarity, which are significant in developing an *in vitro* drug liver toxicity evaluation system. Absorption and metabolism of drugs rely on the highly polarized state of healthy mature hepatocytes. On one hand, hepatocytes are arranged in radial single lines in a plate shape with endothelial cells, called the hepatic plate, and sustain two countercurrent flow systems—the space of Disse (the basal membrane) and bile canaliculus (the apical membrane) (Figure 1A). On the other hand, the extracellular matrix (ECM) provides structural and functional integrity to the hepatic plate (Martinez-Hernandez and Amenta, 1995). In normal liver tissue, fibronectin (FN) and collagenIV are the major ECM distributed in the space of Disse. Cell-cell and cell-ECM interactions and spatial polarity strongly influence gene expression profiles and, therefore, functionality (Schuppan et al., 2001).

To reproduce and maintain hepatocyte polarity *in vitro*, three-dimensional (3D) hepatocyte culture systems have been developed, as they supply cells with a biomimetic environment that aims to simulate *in vivo* conditions (Zhang et al., 2018; Cui et al., 2018; Saheli et al., 2018). The 3D hepatocyte cultures showed more promising outcomes in simulating the morphology and microenvironment of cells *in vivo* than the 2D culture (Fang and Eglen, 2017). Hence, 3D hepatocyte cultures have become a research hotspot in drug liver toxicity evaluation. There are various 3D culture techniques, including multicellular spheroids (Hurrell et al., 2020), hydrogels (Lou et al., 2018), scaffolds (Fuhrer et al., 2018), 3D bioprinting (Yang et al., 2021), organs-on-chips (Low et al., 2021) and microfluidic systems (van Duinen et al., 2015), each with their own advantages. However, simple 3D culture techniques such as multicellular spheroids can’t simulate the physiological structure of hepatic plate. Superior 3D culture techniques such as 3D bioprinting and organs-on-chips are high-cost and need complex operation. In addition, owing to the failure of current *in vitro* 3D culture models in reflecting cholestasis, the liver toxicity of cholestasis-causing drugs, such as statins, has been underrated. Therefore, it is essential to develop a 3D culture models with mature biotransformation and bile secretion abilities.

The carbohydrate polymer sodium alginate (Na-Alg), a component proposed for 3D matrices, has been commonly applied in cell 3D cultures (Beaumont et al., 2021; Miranda et al., 2010; Dvir-Ginzberg et al., 2008). It is a promising biomaterial for the construction of “micro-bioreactors” and “biohybrid organs” owing to its chemical and mechanical stability and biocompatibility. Owing to its permeability for living cells, its hydrated 3D network allows cells to interact with each other (Wang et al., 2009; Li et al., 2006; Zmora et al., 2002; Khalil et al., 2001). In previous studies, we developed a 3D hepatic plate-like Na-Alg hydrogel fiber co-culture model system (3D-P co-culture system) using the C3A cell line and EA. hy926 cell line, with the help of a microfluidic device (Jia et al., 2020). The results showed that the hepatic-specific gene expression profiles and functionalities of hepatocytes in the 3D-P co-culture system were significantly enhanced and hepatocyte polarity was restored, suggesting that the 3D-P co-culture system has the potential to assess hepatotoxicity. Furthermore, transcriptome sequencing analysis proved that the transcripts associated with the bile secretion pathway were enriched when comparing the differentially expressed genes between 3D hepatic plate-like hydrogel fibers and 3D hybrid hydrogel fibers. But more data and analysis related to bile secretion function in 3D hybrid hydrogel fibers is needed. And as a human hepatoma cell line, the C3A cell line is not suitable for toxicity assessments. To evaluate the potential application of the 3D-P co-culture system for readouts of hepatotoxicity of compounds, we built a 3D-P co-culture system using L-02 and EA. hy926 cell lines and the microfluidic device mentioned earlier, and the fabrication process and materials formula used in this study were the same with our previous study. As an immortalized hepatocyte line, L-02 cells have been proven to be suitable for toxicity assessments and bioartificial liver establishment (Kouam et al., 2017; Zhang and Xu, 2018). In this study, we designed four control systems, i.e., two-dimensional co-culture (2D), collagen sandwich co-culture (CS), 3D hybrid hydrogel fiber (3D-H) co-culture systems and primary human hepatocytes (PHHs). By comparing these four systems, we evaluated the effect of the 3D-P co-culture system on cell behavior through the assessments of cell viability, proliferation, hepatocellular functions, and polarity during a prolonged period of up to 28 days and studied the potential of 3D-P co-culture system in assessing hepatotoxicity.

The improved 3D-P co-culture system showed a significant enhancement in bile secretion. Moreover, bile canaliculus-like structure was observed in the 3D-P co-culture system. These results implied that the L-02/EA. hy926 cell 3D-P co-culture system is able to secrete bile and is advantageous in assessing the hepatotoxicity of compounds that could cause cholestasis. To test this inference, we studied the statin hepatotoxicity predictive ability of the 3D-P co-culture system using high-content imaging and analysis (HCA) methods. In summary, considering its high activity level of Cytochrome P450 3A4 metabolism and bile secretion function, the L-02/EA. hy926 cell 3D-P co-culture system can be widely used in hepatotoxicity prediction and assessment in drug development. This novel, low-cost, and efficient system shows promise in screening and DILI evaluation during drug development.

## Materials and Methods

### Establishment of Four Co-Culture Systems

#### Cell Culture

L-02 cells (human immortalized hepatocyte cell line) and EA. hy926 cells (human vascular endothelial cell line) were obtained from the American Type Culture Collection (ATCC) and cultured in Dulbecco’s Modified Eagle Medium (DMEM, C11995500BT; Gibco, Beijing, China) supplemented with 10% (v/v) fetal bovine serum (A3160801; Gibco), 25 U/mL penicillin, and 25 μg/ml streptomycin (PSA, Invitrogen) and incubated at 37°C in a humidified atmosphere with 5% CO_2_.

In each co-culture system, the cell number ratio of the L-02 and EA. hy926 cell lines was 3:1. Cell suspensions of L-02 and EA. hy926 cell lines for cell function and polarity tests were plated into 6-well plates (703001, 801002; Nest, Jiangsu, China) at a density of 8×10^5^ cells/well. For the drug hepatotoxicity test, the cells were plated into 96-well plates (701001; Nest) at a density of 9.99×10^4^ cells/well. The growth of alginate hydrogel fibers on days 1, 3, 7, 14, 21, and 28 was observed using an inverted microscope (CK-2; Olympus, Japan). Primary human hepatocytes were derived from normal tissue adjacent to hepatic hemangiomas at Zhujiang Hospital, Guangzhou (Supplementary Table S1 for detailed information). The Medical Ethical Council of Zhujiang Hospital approved use of PHHs in this study, and informed consent was provided from all patients. Viability of purified hepatocytes was around 90% as determined by Trypan blue (T6146; Sigma-Aldrich, Shanghai, China). The cells were plated on a Matrigel-coated (703001, 801002; Nest) at a density of 8×10^5^ cells/well and cultured in modified transition and expansion medium (TEM). This was based on Advanced DMEM/F12 (12634010; Invitrogen, Shanghai, China) supplemented with N2 and B27 (17502001, 17504044; Invitrogen), 1 mM sodium pyruvate (11360070; Invitrogen), 10 μg/ml ascorbic acid (PHR1008; Sigma-Aldrich) and the following factors: 20 ng/ml HGF, 20 ng/ml EGF (both from Peprotech, NJ, United States), 10 μM Y27632, 3 μM CHIR99021, 1 μM A8301 (all from TargetMol, MA, United States), 1 μM S1P and 5 μM LPA (SC-201383, SC-201053; Santa Cruz, TX, United States).

#### Fabrication of Cell-Incorporating Alginate Hydrogel Fibers

To fabricate 3D hepatic plate-like hydrogel fibers (3D-P), L-02 and EA. hy926 cell lines were independently suspended in a 0.7% (w/v) sodium alginate (71,238; Sigma-Aldrich, MO, United States) solution supplemented with 0.9% NaCl (342920; Sigma-Aldrich), 1% bovine serum albumin (BSA) (A2058; Sigma-Aldrich), and 10 mM HEPES (BH160; Gen-View Scientific, Beijing Dingguo Changsheng Biotechnology Co., Ltd. Beijing, China) at concentrations of 3 × 10^7^ and 1 × 10^7^ cells/mL for L-02 cells and EA. hy926 cells, respectively. The buffer solution contained 10% (w/v) dextran (31392; Sigma-Aldrich), 0.9% NaCl, and 10 mM HEPES. The same solution with a lower concentration of NaCl (0.72%) and 20 mM BaCl_2_ (342920; Sigma-Aldrich) was used as the gelation solution. These cell suspensions and buffer/gelation solutions were continuously introduced into the microchannel using syringe pumps (Harvard Apparatus, MA, United States). The flow rates of the L-02 cell suspension, EA. hy926 cell suspension, buffer solution, and gelation solution were 20, 10, 5, and 100 μl/min, respectively. As for 3D hybrid hydrogel fibers (3D-H), L-02 and EA. hy926 cell lines were suspended in a hybrid manner in the same sodium alginate solution mentioned above.

#### Fabrication of CS Co-Culture System

Ten milligrams of rat tail tendon collagen type I (200,110; Shengyou, Hangzhou, China) for cell culture were blended in 7,900 μl ultrapure water supplemented with 1,000 μl 10 × phosphate-buffered saline (PBS). The pH was neutralized to 6.9–7.1 with NaOH. Each step was conducted on ice under agitation to prevent collagen polymerization. The final collagen concentration was 0.91 mg/ml, and 350 μL of collagen solution was spread on culture plates. DMEM was spread into the wells for 30 min after the polymerization of the gel. Then, the hybrid cell suspension of L-02 and EA. hy926 cell lines was plated on the culture plates that were prepared as described above. Following cell attachment, a second layer of collagen was added.

### Hepatic Function and Polarity Assays

#### Calcein-Acetoxymethyl Ester/Propidium Iodide Staining

The cells were incubated for 10 min with 2 μmol/L calcein acetoxymethyl ester (Calcein-AM) to detect living cells and with a 4.5 μmol/L propidium iodide (PI) homodimer to detect dead cells (C326 and P346; Dojindo Chemical Technology). PI can only enter cells with damaged membranes. After intercalation with DNA, it produced a bright red fluorescent signal (ex/em, 535/617 nm). Images were generated using an epifluorescence microscope (AXIO Vert. A1; Carl Zeiss Meditec AG, Jena, Germany).

#### Test of Albumin and Urea Synthesis and CYP3A4 Enzyme Activity

For albumin and urea synthesis, the amount of total albumin released into the cell culture medium within 24 h on days 1, 7, 14, 21, and 28 was detected by enzyme-linked immunosorbent assay (human albumin ELISA kit; Rayto RT-6100, Shenzhen, China) according to the recommended protocol. After the co-culture systems were incubated with 20 mM ammonium chloride for 24 h, the amount of urea synthesis was measured and analyzed using the Human Urea Assay Kit (S03036; Rayto) according to the manufacturer’s instructions. CYP3A4 enzyme activity was monitored using the CYP3A4 activity assay kit (Fluorometric) (ab211076; Abcam, MA, United States). Briefly, the cells of the four co-culture systems on days 1, 7, 14, 21, and 28 were washed in cold PBS and incubated with 500 μl ice-cold CYP3A4 assay buffer on ice for 5 min. The homogenates were then centrifuged at 15,000 × g for 15 min at 4°C. At the end of the centrifugation period, the detection reagent was added, and luminescence was read with a Veritas luminometer (BioTek ELx 808, VT, USA) using the settings provided by the manufacturer. The results were normalized to the number of viable cells.

#### Localization of Fluorescein in Bile Canaliculus

The functional integrity of the bile canaliculus (BC) formed in the hepatocyte culture system was assessed by the localization of secreted fluorescein in BC. Cells were treated with fluorescein diacetate (FDA) (F7378; Sigma-Aldrich) (0.1 mg/ml) for 45 min and were immediately observed with an epifluorescence microscope AXIO Vert. A1, to visualize the localization of fluorescein.

#### Total Protein Extraction and Western Blotting

On days 1, 7 and 14, cultured cells were lysed in 2x lysis buffer containing 100 mM Tris-HCl (pH 6.8), 2% mercaptoethanol, 20% glycerol, 4% sodium dodecyl sulfate (SDS), phosphatase inhibitors, and a protease inhibitor cocktail (Roche). Solubilized proteins were collected using centrifugation and quantified using a BCA Protein Assay Kit (P0010S; Beyotime). Proteins were separated on SDS-polyacrylamide gel electrophoresis gels and transferred to polyvinylidene difluoride membranes (IPVH00010, Millipore, MA, United States) for immunoblotting with antibodies. The following primary antibodies were used: RXRA (ab125001), OSTA (ab103442) (Abcam) and β-actin (sc-69879; Santa Cruz).

#### RNA Isolation and Quantitative Real-Time RT-PCR

Total RNA was extracted from L-02 and EA. hy926 cells cultured under 2D, CS, 3D-H, 3D-P hydrogel fiber or PHHs conditions using TRIzol reagent (15596018; Thermo Fisher Scientific, Shanghai, China) in accordance with the manufacturer’s instructions. For quantitative reverse-transcription polymerase chain reaction (qRT-PCR), total RNA was reverse-transcribed to cDNA. Next, cDNA was quantified using real-time PCR with SYBR Green qPCR master mix (4309155; Thermo Fisher Scientific). The tested genes included albumin synthesis gene (*ALB*), urea cycle gene (*CPS1*), phase I enzyme genes (*CYP2C9*, *CYP2E1*, and *CYP3A4*), phase II enzyme genes (*UGT1A1*, *UGT1A3*, and *GSTT1*), nuclear receptor genes (*RXRA*), integrins (*ITGA5* and *ITGB1*), small GTPase family genes (*RAC1*, *RAB20* and *RAB43*), microtubule regulator genes (*APC* and *NSF*), and polarity regulator gene (*Par6*). *GAPDH* was used as a reference gene. The primers used in this study are listed in Table 1.

**TABLE 1:** Primers for qRT-PCR.

Gene	Forward primer	Reverse primer
*ALB*	*CAA​AGA​TGA​CAA​CCC​AAA​CCT​C*	*GGA​TGT​CTT​CTG​GCA​ATT​TCA*
*CPS1*	*AAT​GAG​GTG​GGC​TTA​AAG​CAA​G*	*AGT​TCC​ACT​CCA​CAG​TTC​AGA*
*CYP2C9*	*CCA​AAG​AAC​CTT​GAC​ACC​ACT​C*	*AAT​GCC​CCA​GAG​GAA​AGA​GAG*
*CYP2E1*	*ATG​TCT​GCC​CTC​GGA​GTC​A*	*CGA​TGA​TGG​GAA​GCG​GGA​AA*
*CYP3A4*	*GTG​GTG​ATG​ATT​CCA​AGC​TAT​GC*	*TCC​TTG​TTC​TTC​TTG​CTG​AAT​C*
*UGT1A1*	*GGA​ATC​AAC​TGC​CTT​CAC​CA*	*GCA​ATT​GCC​ATA​GCT​TTC​TTC​T*
*UGT1A3*	*GCC​AAC​AGG​AAG​CCA​CTA​TC*	*CAG​CAA​TTG​CCA​TAG​CTT​TC*
*GSTT1*	*GCC​GCG​CGG​AAA​AGA​TGA​AT*	*ATC​TGG​AGG​GCA​ACC​CTT​CT*
*RXRA*	*GGAGGTGAGGGAGGAGTT*	*GCA​TGA​GTT​AGT​CGC​AGA​CAT*
*ITGA5*	*CTG​TGA​CTA​CTT​TGC​CGT​GAA​C*	*GAG​ATG​AGG​GAC​TGT​AAA​CCG​A*
*ITGB1*	*CTG​CGA​GTG​TGG​TGT​CTG​TAA*	*AAG​GCT​CTG​CAC​TGA​ACA​CAT*
*RAC1*	*TGA​AGG​AGA​AGA​AGC​TGA​CTC​C*	*GAT​CGC​TTC​GTC​AAA​CAC​TG*
*APC*	*GGC​AAC​TTC​TGG​TAA​TGG​TCA*	*GAT​GAC​TTG​TCA​GCC​TTC​GAG*
*NSF*	*CCT​ATT​GGC​CCT​CGA​TTT​TC*	*GAA​AGC​GTT​AAG​CAT​TTC​CAT​C*
*RAB20*	*AGT​CCC​AAT​ATG​GAC​GCT​GG*	*TCT​TGG​CGC​TGG​TCT​AAA​G*
*RAB43*	*GCT​ACT​ACC​GCA​GTG​CCA​AT*	*AAT​GTT​GGA​GCC​CGC​ATA​CT*
*PAR6*	*AAG​ATT​TCC​GCC​AGG​TTT​CC*	*ACC​AGG​CGG​GAG​ATG​AAG​AT*
*GAPDH*	*AGC​CAC​ATC​GCT​CAG​ACA​CC*	*ACC​CGT​TGA​CTC​CGA​CCT​T*

#### Immunohistochemistry and Immunofluorescence Analysis

The cells in the four co-culture systems were washed with PBS and fixed in 4% paraformaldehyde (PFA) for 10 min. Furthermore, hydrogel fibers were embedded in paraffin. Sections of hydrogel fibers (3 μm) were prepared for IHC and IF staining. For IHC, the slides were rehydrated and stained for fibronectin (FN, ab32419; Abcam). For IF, slides and fixed cells were permeabilized with 0.1% Triton X-100 for 20 min and washed with PBS. The samples were then incubated with 5% BSA and 0.5% Triton X-(100 in Tris Buffered Salinesolution for 1 h. The following primary antibodies were purchased from Abcam: CYP3A4 ab124921), zonula occludens-1 (ZO-1, ab216880), cluster of differentiation 26 (CD26/DPPIV, ab119346), multidrug resistance-associated protein 2 (ABCC2/MRP2, ab172630), Rac family small GTPase 1 (RAC1, ab33186), and cell polarity regulator (PAR3, ab64646). The samples were incubated with the primary antibodies for 16 h at 4°C and then washed with PBS. The secondary antibodies used for staining were goat anti-mouse IgG conjugated with Alexa Fluor 488 (ab150078; Abcam) and goat anti-rabbit IgG conjugated with Alexa Fluor 555 (ab150117; Abcam). The samples were then incubated with secondary antibodies for 1 h in the dark and washed with PBS. The cells were incubated with 4′,6-diamidino-2-phenylindole (DAPI) stain (Abcam) for 10 min in the dark and then quickly washed with PBS to stain the nucleus. All confocal images were generated using a Leica fluorescence microscope.

#### Transmission Electron Microscope Analysis

The cells in the hydrogel fibers were fixed with 2.5% glutaraldehyde and sliced into ultrathin sections. All images were obtained using a TEM (H-7500; Hitachi, Japan).

### Assessment of Drug-Induced Hepatotoxicity

#### Cell Cytotoxicity Analysis and IC_50_ Analysis

The hepatotoxic drugs used in this experiment included isoniazid (INH, HY-B0329; MedChemExpress (MCE)), troglitazone (TZD, HY-50935; MCE), and amiodarone hydrochloride (AMD, HY-14188; MCE). Metformin hydrochloride (HY-17471A; MCE) is a non-hepatotoxic drug. The statins used in this experiment included atorvastatin hemicalcium salt (ATO, HY-17379; MCE), rosuvastatin calcium (ROS, HY-17504; MCE), simvastatin (SIM, HY-17502; MCE), fluvastatin sodium (FIU, HY-14664A; MCE), and lovastatin (LOV, HY-N0504; MCE). The highest drug concentrations were determined according to the preliminary experimental results. Specifically, hepatotoxic drugs were serially diluted to six concentrations with culture medium and then added to the cell culture well after 2 days of culture. Cell viability was estimated as the percentage of viable cells after exposure to a compound for 24 h and measured using the Cell Counting Kit-8 (Dojindo Chemical Technology, Shanghai Co. Ltd. Shanghai, China). The IC_50_ of each drug scheme was calculated using the Prohibit method with SPSS 23.0 software. Each assay was performed in triplicates. For alanine aminotransferase (ALT), aspartate aminotransferase (AST), and lactate dehydrogenase (LDH) levels, the supernatants were collected after treatment with a drug at different concentrations for 24 and 48 h, and analyzed immediately with commercially available reagents (S03040, S03030, S03034; Rayto) according to the manufacturer’s instructions.

#### Fluorescence Probes Used in Assessment of Drug-Induced Hepatotoxicity

Three additional fluorescence probes were used in this study, apart from Calcein-AM/PI and FDA, mentioned above. Images were obtained using a confocal microscope (Nikon). The cells were incubated for 30 min with 5 μM CellROX (50103ES50; Yeasen Biotechnology (Shanghai) Co. Ltd. Shanghai, China) to detect oxidative stress in them. CellROX gets localized in the cytoplasm and can react with reactive oxygen species (ROS) to turn bright fluorescent (ex/em, ∼ 545/565 nm). The cells were incubated for 30 min with 200 μM Mitotracker (40740ES50; Yeasen) or 15 μM JC-1(HY-15534; MCE) to detect oxidative stress. The cell-permeant MitoTracker^®^ probes contain a mildly thiol-reactive chloromethyl moiety for mitochondrial labeling. Fluorescence intensity depends on the mitochondrial membrane potential (ex/em, ∼579/599 nm). JC-1 exhibits potential-dependent accumulation in the mitochondria, indicated by a fluorescence emission shift from green to red. While red fluorescence indicates a normal mitochondrial membrane potential, green fluorescence reflects an alteration of the latter (ex/em, ∼510/527 nm).

### Statistics

All experiments were performed at least thrice. The results were presented as the mean ± SD. Significance level among the groups at pre-defined time points (day 1, 7, 14) was analyzed by performing Tukey’s method of one-way ANOVA test. Significance level among 3D-H and 3D-P groups at pre-defined time points (day 21, 28) was analyzed by performing a two-tailed unpaired Student’s t-test. Statistical significance was set at *p* < 0.05.

## Results

### Creation of Cell-Incorporating Hydrogel Fibers Using a Microfluidic Device

**FIGURE 2:**
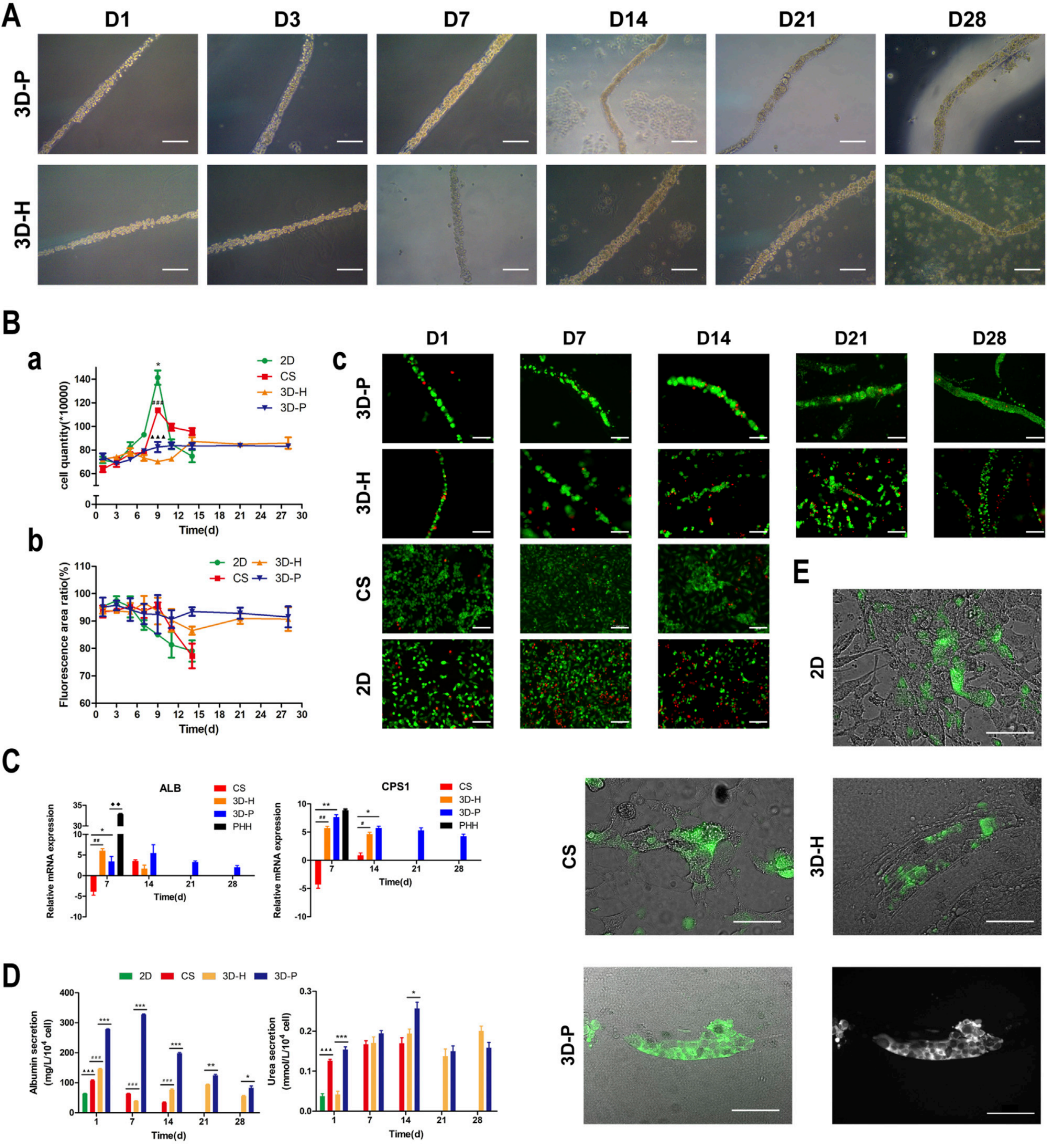
Observation and characterization of 3D hepatic plate-like hydrogel fibers (3D-P). **(A)** Morphological observation of cell-incorporating alginate hydrogel fibers at different time points. **(Ba)** Cell growth curves, **(b)** Cell viability, and **(c)** Calcein-AM/PI staining of four co-culture systems at different time points. **(C)** qRT-PCR analysis of *ALB* and *CPS1* levels, which were normalized to the levels of *GAPDH* and compared to that of cells cultured in 2D co-culture system. The fold changes were log2-transformed. **(D)** Analysis of albumin secretion and urea synthesis of L-02 cells in the four co-culture systems. **(E)** Fluorescent images of the four co-culture systems at day 7 stained with FDA and the grayscale image of 3D-P co-culture system stained with FDA. The arrows indicate the bile canaliculus network. Data are shown as the mean ± SD of triplicate experiments. 3D-P vs. others: *p < 0.05,**p < 0.01, ***p < 0.001; 3D-H vs. others: ^**#**^ *p* < 0.05,^**##**^ *p* < 0.01, ^**###**^ *p* < 0.001; CS vs. 2D:^▲▲▲^ *p* <0.001. PHHs vs. 3D-P: ^◆◆^ *p* < 0.01; 2D: monolayer co-culture, CS: collagen sandwich co-culture, 3D-H: hybrid hydrogel fiber co-culture, 3D-P: hepatic plate-like hydrogel fiber co-culture, PHHs: primary human hepatocytes. Scale bar = 100 μm in **(A,Bc)**. Scale bar = 200 μm in e.

In previous studies, we developed a hydrogel-based cell culture platform to develop 3D hepatic plate-like organoids. In this study, using the same process, we replaced the C3A cell line with the L-02 cell line and assembled it with EA. hy926 cells. Sodium alginate solutions containing L-02/EA. hy926 cells were continuously introduced into a microfluidic channel to produce cell-incorporating Ba-alginate hydrogel microfibers, where L-02 cells at the center were closely sandwiched by EA. hy926 cells (Figure 1A). The results show that the viability of L-02 and EA. hy926 cells after incorporation into the fibers was greater than 95% (Figure 2Bb), indicating that the encapsulation process did not significantly affect cell viability. In the middle stages of culture (7–14 days), plate-like micro-organoids were observed in the 3D-P and 3D-H co-culture systems (Figure 2A). During co-culturing, the average diameters of the hydrogel fibers were 101.8 ± 5.2, 102.4 ± 4.2, and 102.1 ± 4.6 μm on days 1, 14 and 28, respectively, suggesting that 3D hydrogel fibers are replicable and stable (Table 2).

**TABLE 2:** Hydrogel Fiber Diameters in 28 Days of Culture ( $\bar{x}$ ±s, *n* = 3, unit: μm).

	Day 1	Day 7	Day 14	Day 21	Day 28
3D-P	101.8 ± 5.2	102.1 ± 1.7	102.4 ± 4.2	103.1 ± 5.7	102.1 ± 4.6
3D-H	100.5 ± 2.9	102.9 ± 3.6	101.8 ± 5.2	103.2 ± 4.9	103.6 ± 3.6

3D-P, hepatic plate-like hydrogel fiber co-culture system; 3D-H, hybrid hydrogel fiber co-culture system.

### Proliferation and Viability of Cells in 3D-P Co-Culture System

To assess the effect of hepatic plate-like formation on the 3D-P co-culture system, we seeded L-02/EA.hy926 cells in each co-culture system onto 6-well plates at a density of 8 × 10^5^ cells/well for 28 days of culture and observed their morphological changes. Calcein-AM/PI staining was used to measure the cell viability, and the number of cells was counted in the four co-culture systems on days 1, 3, 5, 7, 9, 11, 14, 21, and 28. Cell viability was estimated as the ratio of the fluorescence area of calcein-AM to the total fluorescence area. After culturing for 7 days, hepatocytes started forming aggregates inside the fibers, regardless of the location of EA. hy 926 cells (Figure 2A). The numbers of L-02 and EA. hy926 cells in 3D-P and 3D-H co-culture systems increased gradually for 28 days (Figure 2Ba), and maintained high hepatocyte viability (∼90%) over 28 days (Figures 2Bb,c). By comparison, L-02 cells in the 2D co-culture system under the same conditions and at the same cell seeding density initially grew faster (Figure 2Ba) but reached the proliferative plateau stage on day 9. An increasing number of floating dead cells appeared in the cell culture medium after day 11. With less rapid cell proliferation rates than that of the 2D co-culture system, the CS co-culture system reached the proliferative plateau stage on day 9, and only a small number of floating dead cells was observed by the end of the observation period. Owing to the limited space, after reaching the proliferative plateau stage, the viability of the cells in 2D and CS co-culture systems began to decline on day 6 and day 9, respectively. Owing to the low hepatocyte viability, the observation of 2D and CS co-culture systems ended on day 15.

### 3D-P Co-Culture System Achieved Functional Maturation

The hepatic-specific functions of L-02 cells in different cultures were analyzed by the expression of relevant genes and the quantity of albumin (ALB)and urea produced by L-02 cells. L-02 cells in the 3D-P co-culture system showed greater secretion of ALB and synthesis of urea, compared with that of the other three co-culture systems during the 28-days culture. The secretion levels of ALB in the CS co-culture system showed a slightly declining trend throughout the entire culture period. In the early stages of culture (1–14 days), ALB content in the supernatant of the 3D-P co-culture system was significantly higher than that in the other co-culture systems (*p* < 0.001). Similarly, the urea content synthesized by L-02 cells in the 3D-P co-culture system was higher than that in the 2D and 3D-H co-culture systems on day 1 (*p* < 0.01) (Figure 2D). qRT-PCR analysis indicated that *ALB* and carbamoyl-phosphate synthase 1(*CPS1*) genes showed greater expression in 3D co-cultures, especially in the cells of the 3D-P co-culture system in day 7 and 14, than with L-02 cells under 2D or CS co-culture (*p* < 0.01). The difference of *CPS1* expression level of 3D-P co-culture system and PHHs was not significant in day 7(*p* > 0.05). Nevertheless, the *ALB* expression level of PHHs was significantly higher than that of 3D-P co-culture system (*p* < 0.01) (Figure 2C). Hepatocytes can proactively absorb FDA but can only efflux it *via* transporters, including the bile salt export pump and MRP2. Under 3D culture conditions, the transporters probably polarize expression on the canalicular membrane of cells and are capable of functionally transporting FDA substrates into these canalicular structures. It was observed that abundant retention of FDA within the cell cytoplasm in the 2D co-culture system, as well as the accumulation of the fluorescent substrate in the extracellular space, in CS, 3D-H, and 3D-P co-culture systems. Moreover, in the 3D-P co-culture system, both the area and the fluorescence intensity of the bile canaliculus region were higher, and the fluorescent substrate accumulated in the extracellular space was reticular. These results suggest that the network-like structure was formed in L-02 cells was enhanced in 3D-P co-culture system (Figure 2E).

### Enhanced Metabolic and Biotransformation Competence of L-02 Cells in 3D-P Co-Culture System

**FIGURE 3:**
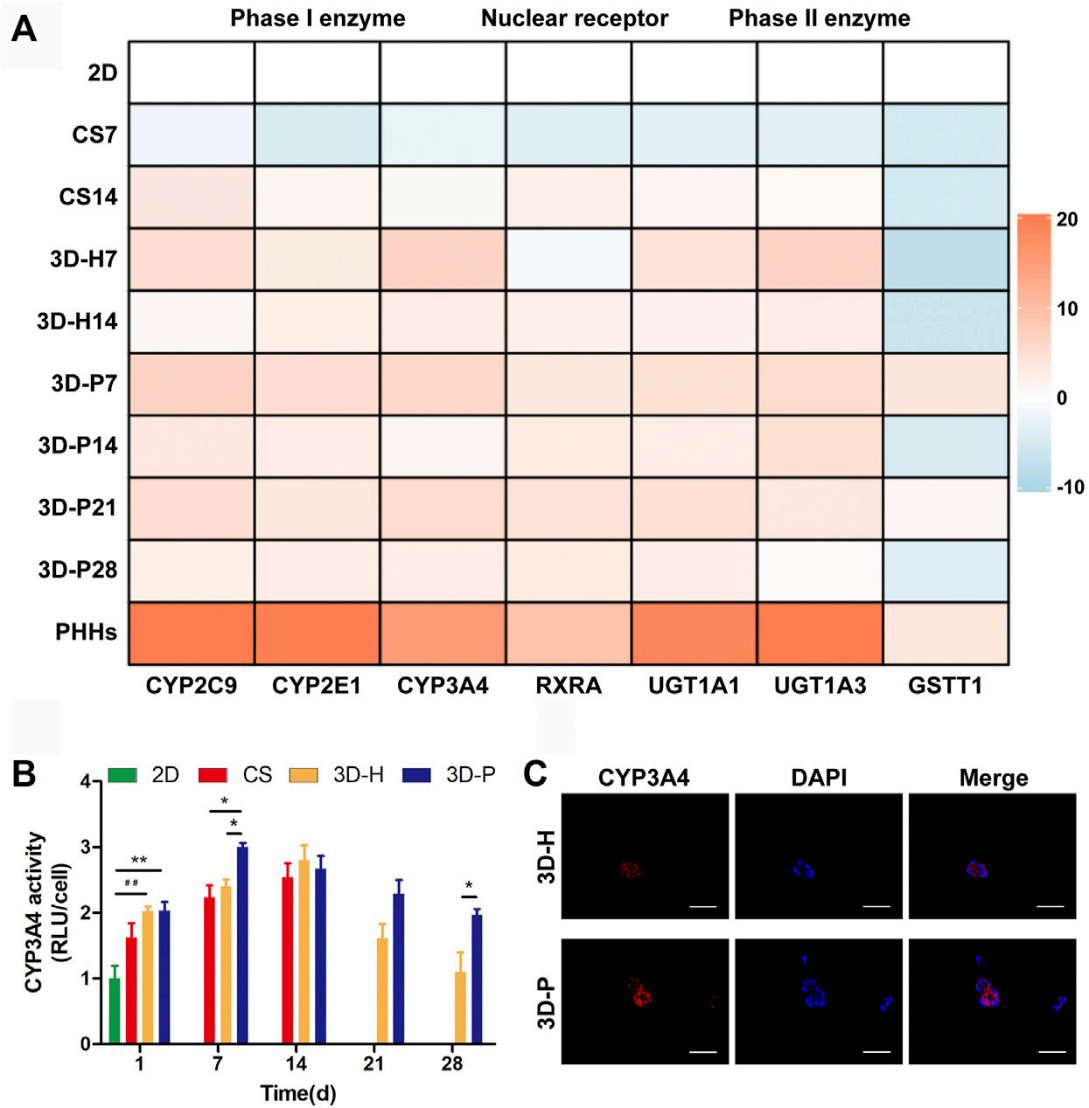
Enhanced metabolic and biotransformation competence of L-02 cells in 3D-P co-culture system. **(A)** Expression of drug metabolism-related genes examined using qRT-PCR. All the gene expression weas normalized to the levels of *GAPDH* and compared to that of cells cultured in the 2D co-culture system. The fold changes were log2-transformed. **(B)** CYP3A4 activities in L-02 cells in the four co-culture systems. The graph represents relative luminescence unit per cell (RLU/cell) with the level of 2D co-cultures defined as 1. **(C)** Immunofluorescence staining of CYP3A4 expression (red) in 3D hydrogel fibers at day 7. Data are shown as the mean ± SD of triplicate experiments. 3D-P vs. others: *p < 0.05,**p < 0.01; 3D-H vs. 2D: ^**##**^ *p* < 0.01. Scale bar = 100 μm.

A series of hepatic-specific xenobiotic and homeostatic metabolism genes were analyzed and compared in L-02 cells under different co-cultures, including serial phase I metabolizing enzymes (*CYP2C9, CYP2E1, CYP3A4*), phase II xenobiotic metabolizing enzymes (*UGT1A1, UGT1A3, GSTT1*), and nuclear receptors (*RXRA*). The expression of hepatic-specific genes was significantly upregulated in the 3D-P co-culture system during the whole culture period, compared to that of the 2D or CS co-culture system for most of the genes tested. In addition, the L-02 cells in the 3D-P co-culture system showed higher expression of all analyzed genes than those in the 3D-H co-culture system in varying proportions (Figure 3A). The 3D-P co-culture system showed a higher level of CYP3A4 metabolism than the 2D co-culture versus 3D-H co-culture system. On day 7, the CYP3A4 activity of 3D-P co-culture system significantly higher than 2D and CS co-culture system (*p* < 0.05) (Figure 3B). Consistent with the above results, IF staining showed greater expression of CYP3A4 in L-02 cells in the 3D-P co-culture system than in the 3D-H co-culture system on day 7 (Figure 3C). There was no difference in *GSTT1* expression level between 3D-P co-culture system and PHHs in day 7 (*p* > 0.05), though the majority of gene expression levels of phase I enzymes, phase II enzymes of PHHs were 3–10 times higher than those of the 3D-P co-culture system generally (Figure 3A).

### Enhanced Hepatocyte Polarity of L-02 Cells in 3D-P Co-Culture System

**FIGURE 4:**
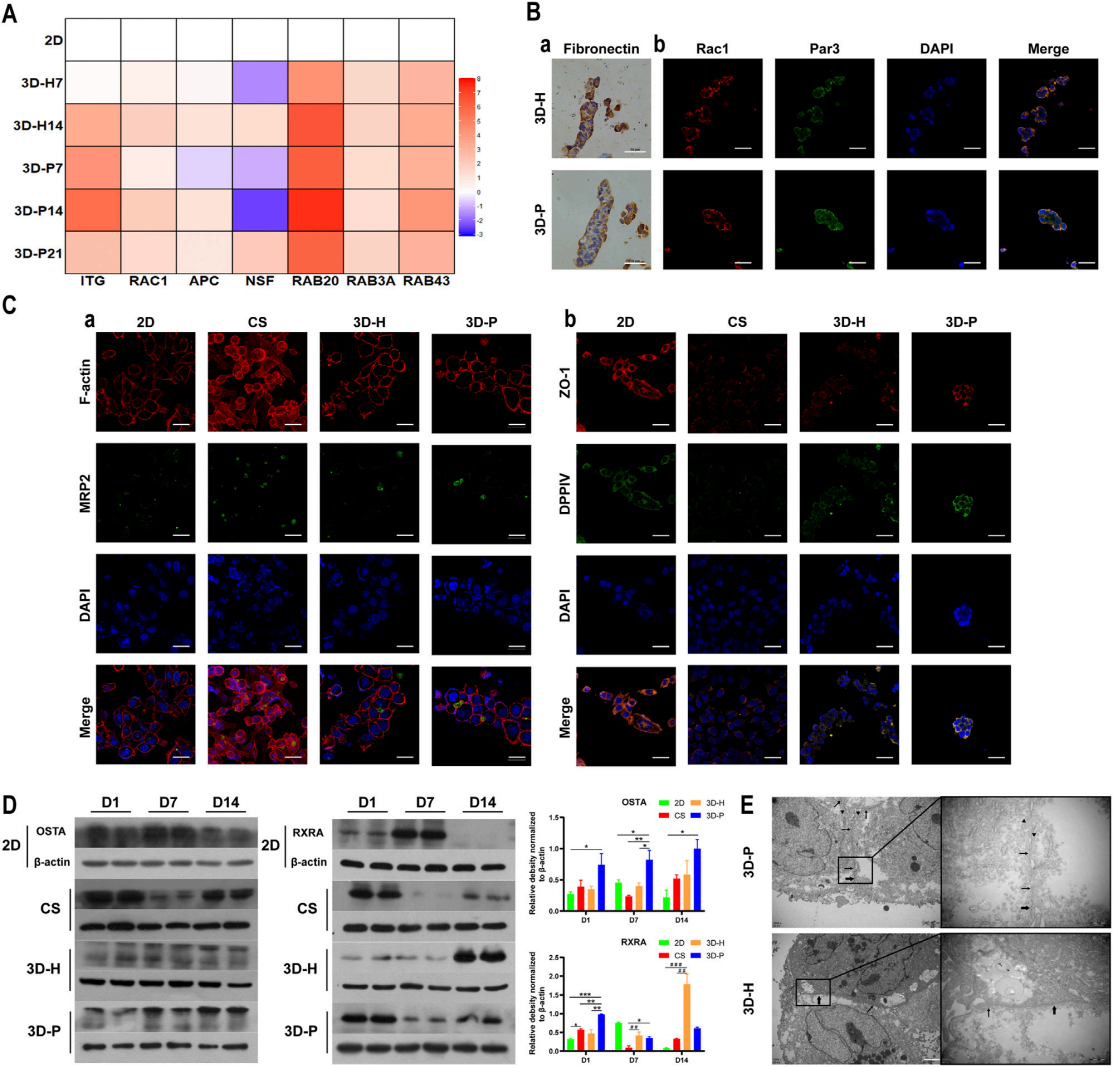
Enhanced polarity of L-02 cells in 3D-P co-culture system. **(A)** qRT-PCR analysis of cell polarity, gene expression which was normalized to the levels of *GAPDH* and compared to that of cells cultured in the 2D co-culture system. The fold changes were log2-transformed. **(B)** IHC staining of **(a)** FN (brown) and IF staining of **(b)** Rac1 (red) and Par3 (green) of the four co-culture systems at day 7. **(C)** IF staining of **(a)** MRP2 (green) and F-actin (red), **(b)** ZO-1 (red) and DPPⅣ (green) of the four co-culture systems at day 7. **(D)** Protein levels of OSTA and RXRA examined using western blotting and were normalized to the levels of β-actin. The relative protein levels were quantified using the ImageJ software. **(E)** TEM images of 3D hydrogel fibers at day 7. The thin and thick arrows indicate the tight and gap junctions between the cells, respectively; the triangular arrows indicate the microvilli. Data are shown as the mean ± SD of triplicate experiments. 3D-P vs. others: *p < 0.05,**p < 0.01; 3D-H vs. 2D: ^**#**^ *p* < 0.05; CS vs. 2D:^▲^ *p* < 0.05. Scale bar = 10 μm in B. Scale bar = 2 μm in C.

To evaluate the hepatocyte polarity of L-02 cells in the four co-culture systems, the expression levels of hepatocyte polarity-related proteins and genes and the ultra-microstructure of cell-cell junctions were monitored. The 3D hydrogel fibers showed markedly higher gene expression levels of the indicator of cell-ECM interactions, integrin (*ITG*), than the 2D culture. Additionally, the gene expression levels of small GTPases (*RAC1*, *RAB20* and *RAB43*), adenomatous polyposis coli (*APC*), and N-ethylmaleimide-sensitive factor 2 (*NSF2*) significantly increased in L-02 cells in the 3D-P co-culture system, when compared with that of the 3D-H co-culture system (Figure 4A). The results were further confirmed by IHC staining of FN, as well as IF staining of Rac1 and apical polarity proteins, Par3. It could be detected that in the 3D-P co-culture system, FN was mainly located on both sides of the liver plate-like microstructure, which was disorderly distributed in 3D-H co-culture system. Similarly, IF staining of Rac1 and Par3 showed the polarity of the hepatocytes in 3D-P co-culture system. Rac1 was mainly located at the periphery of the liver plate-like microstructure and Par3 was mainly located on the cell membrane between hepatocytes (Figure 4B). In addition, The results were also further confirmed by IF staining of the cytoskeleton (F-actin), apical polarity proteins (MRP2), tight junction proteins (ZO-1), and luminal polarity protein (CD26/DPPIV) in the four co-culture systems (Figure 4C), as well as by the protein expression levels of sodium/bile acid cotransporters (OSTA) and nuclear receptor (RXRA) (Figure 4C). In the 3D-P co-culture system, the protein levels of OSTA increased significantly in all stages (*p* < 0.05), especially in the middle stages, on the other hand, the protein expression levels of RXRA were significantly increased in the early stages (*p* < 0.01). Interestingly, more canaliculi, marked by green fluorescence of MRP2, were observed in 3D-P hydrogel fibers versus 3D-H hydrogel fibers, and their forms were more regular than those of the 2D co-culture system (Figure 4Ca). The ultra-microstructure in TEM images showed cell-cell junctions in 3D hydrogel fibers, including tight junctions, gap junctions, and microvilli. More microvilli could be found in the 3D-P co-culture system, which proves that the structure of the canaliculi in the 3D-P co-culture system was more mature than that in the 3D-H co-culture system (Figure 4E).

### 3D-P Co-Culture System can be Used for Assessment of Drug-Induced Hepatotoxicity

**FIGURE 5:**
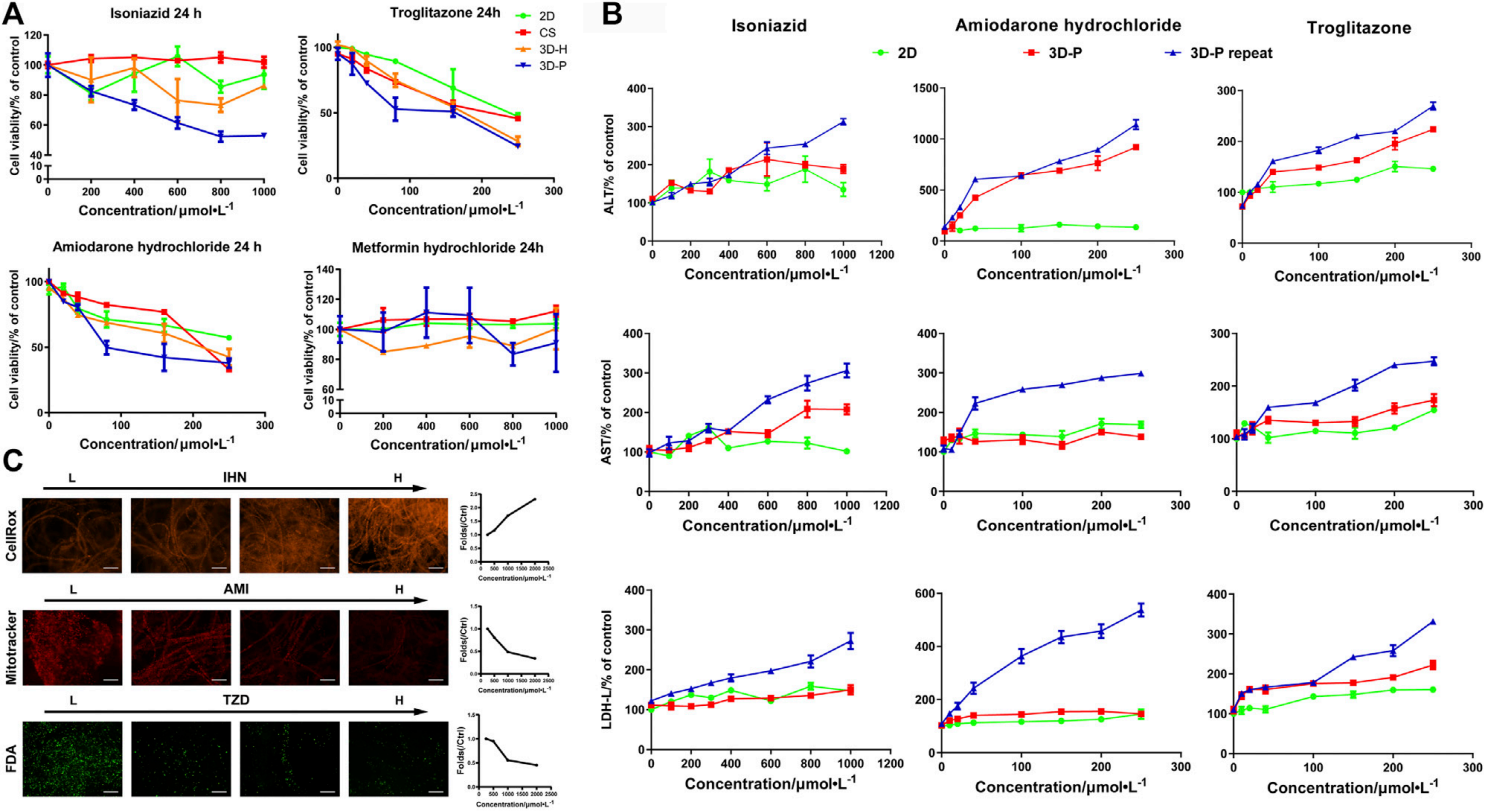
Use of 3D-P co-culture system in the assessment of drug-induced hepatotoxicity. **(A)** Cytotoxicity analysis of L-02 cells in the four co-culture systems with treatment of hepatotoxic (troglitazone (TZD), amiodarone hydrochloride (AMD), and isoniazid (INH)) or non-hepatotoxic drugs (metformin hydrochloride) **(B)** Assessments of ALT, AST, and LDH levels in L-02 cells at 2D and 3D-P co-culture systems with a single treatment of hepatotoxic drugs (INH, AMD, TZD) (24 h) or repeated treatments (7 days). **(C)** Multicolor cellular images obtained from HCA assessments on 3D-P co-culture system treated with the hepatotoxic drugs and the dose-dependent AFI curves. All values are presented as normalized relative to control. The data are shown as the mean ± SD of triplicate experiments. Scale bar = 100 μm for C.

To study the practical application of the 3D-P co-culture system in assessing DILI, cell responses to several known hepatotoxic drugs were assessed in single and repeated doses, which enabled us to observe reactions to short-term and long-term exposures. The reported non-hepatotoxic compound, metformin hydrochloride, showed no toxicity to cells in all co-culture systems (Figure 5A). By contrast, the viability of L-02 cells in the 3D-P co-culture system treated with INH, AMD or TZDs, commonly known for their hepatotoxicity, significantly decreased. The results indicated that amiodarone hydrochloride and troglitazone may cause dose-dependent hepatic injury in the four co-culture systems. Interestingly, isoniazid only decreased the viability of L-02 cells in the 3D-P co-culture system. Furthermore, the IC_50_ values of the hepatotoxic compounds the 3D-P co-culture system was treated with, were lower than those in the other three co-culture systems (Table 3), suggesting that the 3D-P co-culture system was more sensitive to hepatotoxic compounds, owing to the greater activity of CYP3A4. The release of ALT, AST, and LDH enzymes is an important index for assessing liver injury. After a single treatment with INH, AMD, or TZD at the same concentration, the ALT, AST, and LDH release from the 3D-P co-culture system significantly increased, compared with that of the 2D co-culture system. Similarly, after repeated drug exposures, the amount of enzymes released in the 3D-P co-culture system was 3–10 times that in the 2D co-culture system (Figure 5B).

**TABLE 3:** Half maximal inhibitory concentrations [IC_50_(nmol/L)] of tested drugs.

	2D	CS	3D-H	3D-P
INH	—	—	—	1,031
AMD	181	104	98	73
TZD	232	119	130	114
Metformin	—	—	—	—

TZD, troglitazone; AMD, amiodarone hydrochloride; INH, isoniazid; −, negative.

There are multiple mechanisms of hepatocyte injury initiated by different drugs, such as hepatocellular lesions, fibrosis, necrosis, steatosis, cholestasis, or their combinations. To assess the hepatotoxic pathways of INH, AMD or TZDs, the average fluorescence intensity (AFI) of cellular images of three key cell events were analysis. The 3D-P co-culture system treated with INH showed an increase in the AFI of CellROX in a dose-dependent manner. Similarly, the 3D-P co-culture system treated with AMD showed a dose-dependent decrease in AFI of Mitotracker. The TZD-treated 3D-P co-culture system stained with FDA showed a significantly weakened bile excretion and AFI in a dose-response manner (Figure 5C), which prompted the inhibition of hepatic bile acid-competing transport pathways. Thus, the 3D-P co-culture system faithfully reflected the cytotoxicity of known hepatotoxicants and their possible mechanisms such as oxidative stress, mitochondrial damage, and cholestasis.

### Evaluation of Statin-Induced Hepatotoxicity in 3D-P Co-Culture System

**FIGURE 6:**
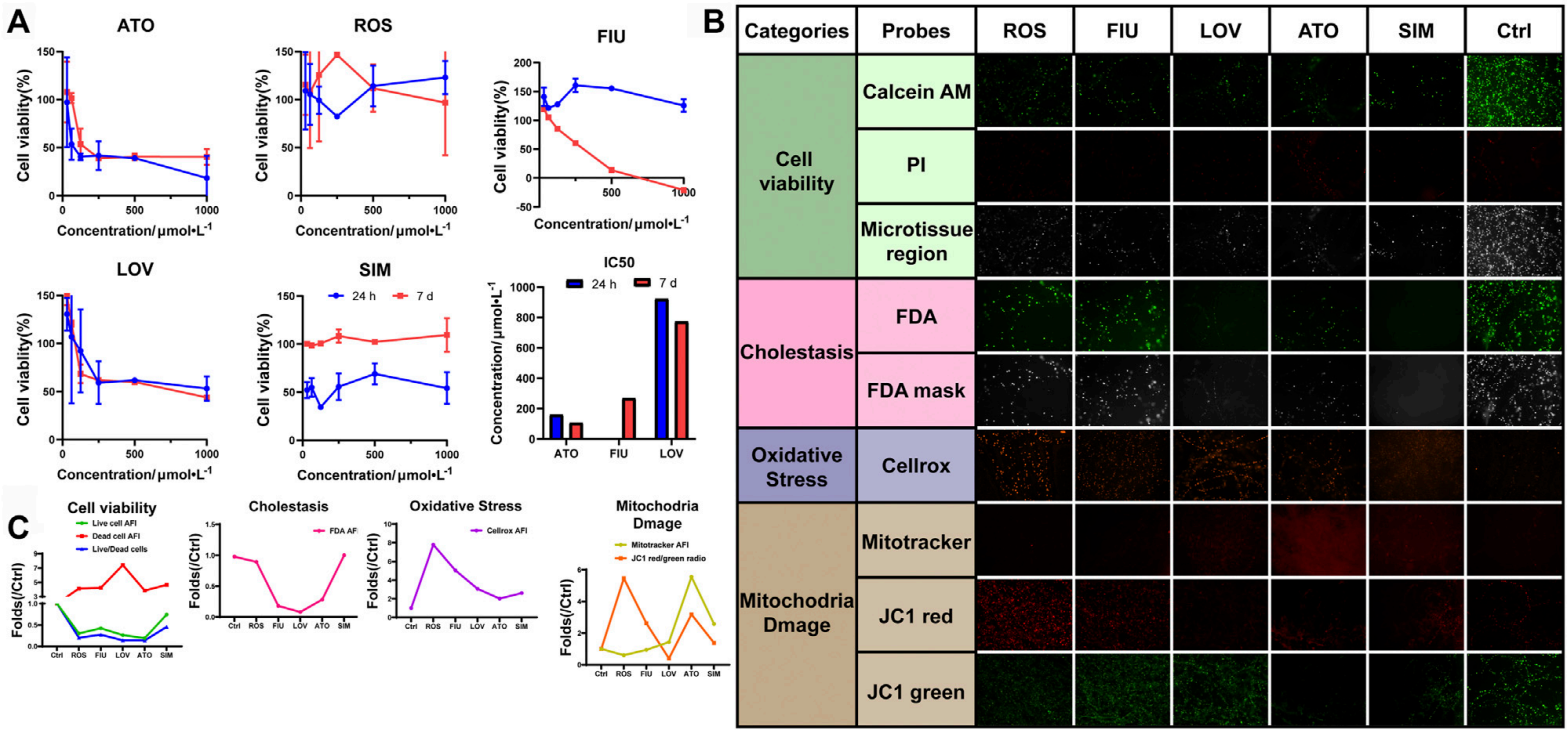
Use of 3D-P co-culture system in the assessment of statin-induced hepatotoxicity. **(A)** Cytotoxicity and IC_50_ analyses of L-02 cells in the four co-culture systems with treatment of five statins (ATO: atorvastatin; ROS: rosuvastatin; FIU: fluvastatin; LOV: lovastatin; SIM: simvastatin; control: 0.1% DMSO). Multiparametric HCA assessments of 3D-P co-culture system at day 7 exposed to different statins for 24 h: ATO (100 μmol/L); ROS (100 μmol/L); FIU (300 μmol/L); LOV (1000 μmol/L); SIM (100 μmol/L). After incubation, seven fluorescence-based endpoints were measured to assess chemically induced alterations in cellular functions. **(B)** Representative confocal images shown correspond to each drug. **(C)** Image analysis readouts were derived from the analysis performed in 3D space. All values are presented as normalized relative to the control. The data are shown as the mean ± SD of triplicate experiments. Scale bar = 100 μm for B.

Hyperlipidemia has become a significant public health problem worldwide in recent decades. Hydroxymethylglutaryl-coenzyme A reductase inhibitors (statins) are commonly prescribed drugs for the treatment of hyperlipidemia. However, even at therapeutic doses, statins may induce an increase in aminotransferase levels. We used the 3D-P co-culture system to evaluate hepatotoxicity induced by five different statins, classified into four categories, namely, cell viability, cholestasis, oxidative stress, and mitochondrial damage. The cytotoxicity profiles revealed that the 3D-P co-culture system was not only suitable for evaluations of short-term and single drug-induced hepatotoxicity but also for long-term and repeated drug-induced hepatotoxicity (Figure 6A). The IC_50_ levels of each drug showed that atorvastatin and lovastatin exhibited both short-term and long-term drug-induced toxicity, compared with other drugs (Figure 6A). By contrast, rosuvastatin and simvastatin caused low cytotoxicity in L02 cells in the 3D-P co-culture system. Interestingly, fluvastatin showed long-term hepatotoxicity instead of short-term. To assess the potential pathways of statin-associated hepatotoxicity, multiparametric analysis of the 3D-P co-culture system treated with different statins was performed. It was stained with selected fluorescence probes and monitored using hyper-multicolor cellular imaging after exposure to five statins for 24 h. Based on the images obtained (Figure 6B), the specificity of each high-resolution cellular imaging parameter was quantified using ImageJ and normalized to the findings in the controls in a dose-dependent manner (Figure 6C). Three out of the five drugs (including fluvastatin, lovastatin, and atorvastatin) induced cholestasis with decreasing fluorescence intensity in the bile canaliculus and decreasing area of the bile region. One of them, lovastatin, significantly caused mitochondrial dysfunction in cells, as indicated by the decreased AFI of MitoTracker and JC-1 red/green ratio. The increased ROS production induced by lovastatin, atorvastatin, and simvastatin was consistent with known biological changes due to oxidative stress. These results demonstrated that the 3D-P co-culture system has the potential to predict drug-induced hepatotoxicity with high specificity, as well as assist in understanding the active mechanisms of hepatic injury.

## Discussion

Here, we established a 3D hepatic hydrogel fiber co-culture system with a hepatic plate-like structure prepared from L-02/EA.hy926 cell lines. Our results showed that the gene expression of the RHO family members, small GTPases, as well as various hepatocyte membrane proteins and apical polarity proteins were significantly increased in L-02 cells in 3D co-culture systems (particularly in the 3D-P co-culture system), when compared with that of the 2D co-culture system. The IF staining analysis showed that proteins specifically expressed on the basal membrane (OSTA, DPPIV) and the apical membrane (MRP2, Par3) were highly expressed in the 3D-P co-culture system, indicating that the hepatocytes in the 3D-P co-culture system were maintained in a polarized state during the long-term culture. Moreover, the albumin secreting and urea producing capacity of the L-02 cells in the 3D-P co-culture system was consistently higher than that in the other co-culture systems. Gene expression analysis showed that the L-02 cells in the 3D-P co-culture system expressed most of the genes related to drug metabolism, such as of phase I and II enzymes, as well as nuclear receptors. The significantly increase of the gene and protein expression of RXRA contributes to the high expression level of CYP3A4. It has been reported that RXRA can form heterodimers with the pregnane X receptor (PXR) and bind to multiple elements in CYP3A promoters *in vitro* and activate the CYP3A promoter in cells (Kliewer et al., 1998; Goodwin et al., 1999). In addition, the CYP3A4 activity of the L-02 cells in the 3D-P co-culture system was maintained at a high level during 28 days of continuous culture. Our previous studies found that 3D-P co-culture system could influence the bile secretion pathway, which is important in regulating drug metabolism (Willson and Kliewer, 2002). In this study, we found that, the expression levels of ZO-1 and MRP2, which are regarded as markers of bile canaliculus formation (Treyer and Musch, 2013; Gissen and Arias, 2015), were significantly increased in 3D-P co-culture system. Moreover, reticular fluorescent substrates and mature microvilli was observed in 3D-P co-culture system. The results suggested that mature bile canaliculus were formed in 3D-P co-culture system and it might be a good *in vitro* model for hepatotoxic drug screening.

The function advantages of the 3D-P co-culture system over the 3D-H co-culture system may attributed to the significant influence on the physical characteristics of hepatocytes. The Na-Alg hydrogel medium played a fundamental role in the polarization of hepatocytes in the 3D-P co-culture system. The Na-Alg hydrogel provides an appropriate mechanical strength mimicking the ECM scaffold. Earlier, hepatocyte proliferation was restricted in 3D hepatic hydrogel fibers, probably because of the Na-Alg hydrogel peridium and contact inhibition. By restricting hepatocyte proliferation and providing a hydrated 3D network that allows cells to interact with each other, the Na-Alg hydrogel medium provides appropriate initial conditions for hepatocyte polarization. However, the Na-Alg hydrogel medium on their own are insufficient to instruct cells to form polar tissue structures. Wrapped by the Na-Alg hydrogel medium, the FN secreted by the EA. hy926 cell line induced an anisotropic intercellular mechanical tension which is crucial in inducing hepatocyte polarization. It has been reported that ECM can be contained in Na-Alg hydrogel and influence cell polarity (Lamaison et al., 2021; Doméjean et al., 2016), which is consistent with our findings. In the 3D-P co-culture system, FN distributed on both sides of hepatocytes and might induce polar intercellular mechanical tension and further guide the formation of bile canaliculus by mimicking the ECM in the space of Disse, which will not occur in most of the nonpolar 3D co-culture systems. By inducing an anisotropic intercellular mechanical tension and bind with integrin, ECM scaffolding could induce the activity of small GTPases such as Rac1(Sigurbjornsdottir et al., 2014), guide lumen elongation, as well as maintain its tight structure (Li et al., 2016) (Figure 1B). This may explain enhancement of the polarity and formation of bile canaliculus in the 3D-P co-culture system and the its long term maintenance.

One of the major shortcomings of current drug safety assessment studies is the failure of existing *in vitro* models to adequately reflect hepatotoxic responses. This failure was thought to be related to the low activity of cytochrome P450 (CYP450) enzymes in hepatocytes. The first step in the metabolism of compounds is completed by phase I metabolizing enzymes, which can introduce small chemical changes that facilitate compound excretion. As a major CYP450 enzyme, CYP3A4 functions in the biotransformation of more than 50% of all prescription medications (Delafuente, 2003). Therefore, the ability to maintain a high activity of CYP450 enzymes is considered the basis for constructing an *in vitro* model for drug safety assessments. Also, many xenobiotics are nontoxic, unless they are converted into reactive intermediates by phase I metabolizing enzymes. For example, as the treatment of type 2 non-insulin-dependent diabetes mellitus, TZD was withdrawn in the year 2000 because of hepatotoxicity (Yokoi, 2010). TZD is metabolized *via* CYP2C9 and CYP3A4 to a quinone metabolite, which induces oxidative stress (Scheen, 2007) and lead to intrahepatic cholestasis (Ogimura et al., 2017). Antituberculosis drugs have also caused DILI in mainland China. As the main antituberculosis drug, isoniazid is metabolized by hepatic N-acetyltransferase (NAT) and CYP2E1 to form hepatotoxins (Huang et al., 2003). Our results showed that the gene expression level and the functional activity of CYP3A4 significantly increased in the 3D-P co-culture system, with a tendency to stabilize after day 14. Our results implied that INH can cause oxidative stress which were identical to those reported in literature (Ramachandran et al., 2018). Increased activity of the CYP450 enzymes enhanced the metabolism of the hepatotoxins, which may explain why the C showed the highest sensitivity of drug-induced toxicity when compared with the other co-culture systems. However, some xenobiotics are directly hepatotoxic. The mitochondrial toxicity of amiodarone hydrochloride, a highly effective antiarrhythmic agent, is triggered by its benzofuran structure (Spaniol et al., 2001; Sung and Yoon, 2012). Interestingly, the 3D-P co-culture system also showed the highest sensitivity of its toxicity when compared with the other co-culture systems. In summary, the 3D hepatic plate-like hydrogel fiber co-culture system can sensitively reflect the dose-dependent hepatotoxicity of these known hepatotoxic drugs. In summary, the 3D-P co-culture system was able to sensitively identify the cytotoxicity of hepatotoxicants to evaluate DILI.

The other major shortcomings of current *in vitro* models is the insufficient bile secretion function. The bile canaliculus is essential in hepatic drug metabolism and cholestasis is one of the most common mechanisms of DILI. Statins are known to induce both acute and chronic liver injury (Clarke and Mills, 2006; Chalasani et al., 2008) and cholestasis (Björnsson et al., 2012). The increasing incidence of hyperlipidemia and coronary artery disease has led to their wide usage. Therefore, it is essential to develop an *in vitro* 3D hepatocyte culture system with an active bile secretion function and which establishes a DILI predicting system by combining the HCA methods. In our study, the enhancement of bile secretion function and the observation of bile canaliculus network indicate that the 3D-P co-culture system may be an appropriate model for hepatotoxicity evaluation of compounds that may cause cholestasis.

Five statins were analyzed using the proposed system. We carried out short-term (single-dose) and long-term (repeated-dose) experiments and investigated their toxicity using cell viability analysis. Three statins showed short-term toxicity, and two showed long-term toxicity. We wished to examine the prospects of using *ex vivo* studies compared to the costlier *in vivo* ones, to assess drug hepatotoxicity with HCA to analyze the 3D-P co-culture system. For this purpose, five statins were evaluated using HCA. After treatment with the drugs, the 3D-P co-culture system was stained and used to generate images using a confocal microscope. The drug-induced alterations in the 3D-P co-culture system were analyzed with respect to the four parameters, namely, cell viability, cholestasis, oxidative stress, and mitochondrial damage. We learn that different statins induce hepatotoxicity *via* different mechanisms. For example, atorvastatin might cause cholestatic liver injury with long-term treatment, which corresponds to previous reports (Clarke and Mills, 2006) fluvastatin has been associated with acute DILI (Castiella et al., 2007), and no cases of chronic injury have been reported, which was further confirmed by our results. Thus, with the help of a simple visual system based on staining results, we can predict the safety and hepatotoxicity of the drug and the possible mechanisms by which it may cause either acute or chronic DILI.

There are still some limits in this work. The gene expression levels of phase I enzymes and phase II enzymes in the L-02 cells were lower than that in PHHs, except for *CPS1, CSTT1*, which is a common defect of hepatocyte cell lines. Furthermore, the mechanisms of forming bile secretion and the bile canaliculus network-like structure in 3D-P co-culture system need to be further studied.

## Conclusion

We established a 3D co-culture system with hepatic plate-like formation, prepared from L-02/EA.hy 926 cells. This formation gives the system advantages of cell polarity and liver-specific functions, especially drug metabolism and bile secretion. Moreover, the 3D-P co-culture system was capable of predicting and evaluating acute or chronic DILI. In the near future, to make this system more comprehensive and reliable, it can be upgraded by mixing multiple types of cells, such as immune cells and hepatic stellate cells to support predictions of other kinds of DILI, such as autoimmune-like DILI.

## Data Availability

The datasets presented in this study can be found in online repositories. The names of the repository/repositories and accession number(s) can be found in the article/Supplementary Material.
