# Supplementary material for: Application of 3D Hepatic Plate-Like Liver Model for Statin-Induced Hepatotoxicity Evaluation
Source: Front Bioeng Biotechnol. 2022 Mar 17;10:826093. doi: 10.3389/fbioe.2022.826093 (PMC8968918; doi:10.3389/fbioe.2022.826093)
Supplement: Supplementary file 1 [file DataSheet1.PDF]

Supplementary Table S1 Donor information.

Identifier	Gender	Age	Associated pathology
Donor 1	Male	52	Hepatic hemangioma
Donor 2	Male	48	Hepatic hemangioma
Donor 3	Female	50	Hepatic hemangioma
